# High Mobility Group Box-1 and Blood–Brain Barrier Disruption

**DOI:** 10.3390/cells9122650

**Published:** 2020-12-10

**Authors:** Masahiro Nishibori, Dengli Wang, Daiki Ousaka, Hidenori Wake

**Affiliations:** Department of Pharmacology, Okayama University Graduate School of Medicine, Dentistry and Pharmaceutical Sciences, Okayama 700-8558, Japan; wangdengli.cool@163.com (D.W.); daiki_ouou@hotmail.co.jp (D.O.); wake-h@cc.okayama-u.ac.jp (H.W.)

**Keywords:** high mobility group box-1, blood–brain barrier, inflammation, stroke, trauma, vascular endothelial cell, pericyte, monoclonal antibody

## Abstract

Increasing evidence suggests that inflammatory responses are involved in the progression of brain injuries induced by a diverse range of insults, including ischemia, hemorrhage, trauma, epilepsy, and degenerative diseases. During the processes of inflammation, disruption of the blood–brain barrier (BBB) may play a critical role in the enhancement of inflammatory responses and may initiate brain damage because the BBB constitutes an interface between the brain parenchyma and the bloodstream containing blood cells and plasma. The BBB has a distinct structure compared with those in peripheral tissues: it is composed of vascular endothelial cells with tight junctions, numerous pericytes surrounding endothelial cells, astrocytic endfeet, and a basement membrane structure. Under physiological conditions, the BBB should function as an important element in the neurovascular unit (NVU). High mobility group box-1 (HMGB1), a nonhistone nuclear protein, is ubiquitously expressed in almost all kinds of cells. HMGB1 plays important roles in the maintenance of chromatin structure, the regulation of transcription activity, and DNA repair in nuclei. On the other hand, HMGB1 is considered to be a representative damage-associated molecular pattern (DAMP) because it is translocated and released extracellularly from different types of brain cells, including neurons and glia, contributing to the pathophysiology of many diseases in the central nervous system (CNS). The regulation of HMGB1 release or the neutralization of extracellular HMGB1 produces beneficial effects on brain injuries induced by ischemia, hemorrhage, trauma, epilepsy, and Alzheimer’s amyloidpathy in animal models and is associated with improvement of the neurological symptoms. In the present review, we focus on the dynamics of HMGB1 translocation in different disease conditions in the CNS and discuss the functional roles of extracellular HMGB1 in BBB disruption and brain inflammation. There might be common as well as distinct inflammatory processes for each CNS disease. This review will provide novel insights toward an improved understanding of a common pathophysiological process of CNS diseases, namely, BBB disruption mediated by HMGB1. It is proposed that HMGB1 might be an excellent target for the treatment of CNS diseases with BBB disruption.

## 1. Introduction

The brain receives 15% of blood flow from cardiac output and consumes 20% of the total supply of oxygen from blood, although the wet weight of the brain is only 2% of body weight. Blood flow and oxygen consumption in the brain are the highest among the organs because brain tissue uses most of its energy to continuously maintain the intra/extracellular ion environment through ATP-dependent ion transport, especially in neurons [1]. This feature allows the brain to maintain the integrity and activity of the neural network. Cerebral blood flow (CBF), oxygen delivery, and energy metabolite supply should be coordinated with neural activity by integrating the demands from neuronal and glial elements. A recent study on the physiological and pathophysiological processes involved in the control of brain functions and relevant disorders introduced the concept of the “neurovascular unit” (NVU) to help model the intimate relationship between the neural and vascular systems [1,2,3].

**Figure 1:**
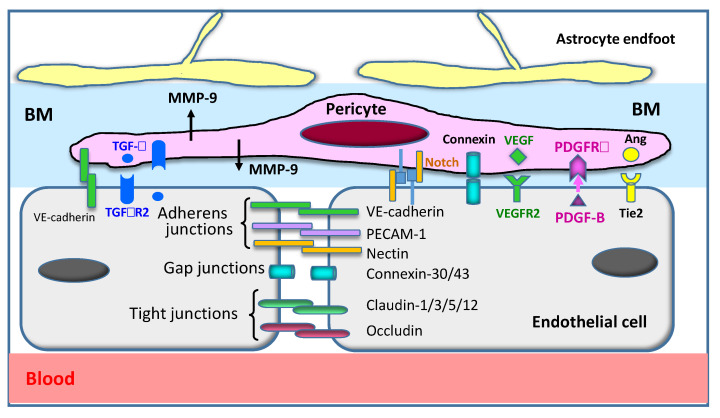
Structure of the blood–brain barrier (BBB). The BBB consists of vascular endothelial cells, pericytes, a basement membrane (BM), and astrocyte endfeet. Cellular components are integrated with many adhesive molecules and are functionally regulated by ligand–receptor systems. The major factors are drawn in this figure. Ang: angiopoietin; BM: basement membrane; MMP-9: matrix metallo proteinase-9; PECAM-1: platelet endothelial cell adhesion molecule-1; PDGF-B: platelet-derived growth factor-B; PDGFRb: platelet-derived growth factor receptor beta; TGF-b: transforming growth factor-b; TGFbR2: Transforming growth factor-b receptor 2; VE-cadherin: vascular endothelial cadherin; VEGF: vascular endothelial growth factor; VEGFR2: vascular endothelial growth factor receptor 2.

The blood–brain barrier (BBB) is a distinctive structure of capillary vessels in the brain [4,5,6]. The BBB, which is composed of vascular endothelial cells, pericytes, extracellular matrix, and endfeet of astrocytic processes, functions as a barrier against the entry of microorganisms, toxins, bioactive substances, and many kinds of solutes, including drugs (Figure 1) [2,3,6,7]. The BBB also limits the improvident infiltration of white blood cells into the brain parenchyma. On the other hand, transporter systems exist in each component of the BBB to transport a diverse range of substances that are necessary for the maintenance of brain function [3,8,9,10]. It is well known that the capillaries in the brain have higher numbers of pericytes compared with the capillaries in peripheral vasculatures [7], and pericytes may play an important role in maintaining the integrity of the BBB, the contractility of capillaries, and the development and maturation of this structure [3,11,12,13].

Recent studies have demonstrated that dysfunction or disruption of the BBB may be involved in many diseases, including brain infarction [14,15], hemorrhage [3,16], trauma [17,18,19], and epilepsy [20,21,22,23], as well as neurodegenerative diseases [3,24,25]. Disruption of the BBB appears to be intimately associated with brain inflammatory responses [1,3,25], and the infiltration of immune cells through the dysregulated BBB [26] may enhance the inflammation inside the brain parenchyma in collaboration with the activated residual glial cells [5,27,28]. Thus, the BBB functions as the interface between circulating inflammatory and immune cells and brain parenchymal cells, and, therefore, functional regulation and maintenance of the BBB are very important for strictly controlling the maintenance and protection of brain physiology [27].

High mobility group box-1 (HMGB1) is a highly conserved nonhistone nuclear protein that contributes to the architecture of chromatin DNA and regulates the transcriptional activity of genes. A neurite-promoting factor, amphoterin, purified from perinatal rat brain [29,30], was found to be identical to HMGB1 [30,31]. HMGB1 was also reported to be a late mediator of endotoxemia in mice [32] and has been shown to function as a representative damage-associated molecular pattern (DAMP), promoting inflammatory responses in many disease conditions [25,33,34,35]. In an earlier study, HMGB1 was reported to be released from necrotic cells [36], but soon thereafter, it was recognized that HMGB1 may be released from living cells as well under different stress conditions, including hypoxia, ischemia, and stimulation by cytokines [25,33,37]. Therefore, HMGB1 appears to be a sensor molecule that responds to a diverse range of stimuli. Once released into extracellular space, HMGB1 stimulates multiple receptors, including RAGE and TLR-4/2 [33]. In addition, HMGB1 may bind to IL-1a, IL-1b, CXCL12, DNA, RNA, and LPS when these molecules coexist with HMGB1, leading to enhanced activation of the cognate receptors of each factor [37,38]. HMGB1 has also been reported to exhibit a chaperone-like function, by which it carries LPS and nucleic acids into cells [39,40]. This diverse range of functional modes may characterize HMGB1 as a proinflammatory molecule.

There is increasing evidence suggesting the involvement of BBB disruption in many kinds of brain diseases, including brain infarction, hemorrhage, trauma, epilepsy, and neurodegenerations [3,24,25]. BBB disruption significantly contributes to brain inflammation through the leakage of plasma factors into the brain, the blocking of endothelial–pericyte interaction, the activation of glial cells, and the induction of immune cell migration into brain tissue [3,26,41]. Conversely, brain inflammation facilitates BBB disruption through digestion of the basement membrane by proteinases and the activation of lesions of component cells [41,42,43]. These mutual events may lead to brain edema, hypoxia, excitatory amino acid release, and continued elevation of calcium in neurons, and eventually to neuronal death [3,25].

In this review, we will focus on the breakdown and disruption of the BBB under different conditions, including brain infarction, brain hemorrhage, brain trauma, epilepsy, degenerative diseases, and systemic inflammation, and we will discuss the involvement of released HMGB1 in the processes. We propose that the direct neutralization of extracellular HMGB1 by a monoclonal antibody (mAb) or the inhibition of HMGB1 release by small molecules may be a novel strategy for the treatment of neuronal injuries induced by different insults.

Restriction of the penetration of a diverse range of substances into the brain is the fundamental function of the BBB [3,4,6]. The BBB is composed of vascular endothelial cells, pericytes, endfeet of astrocytes, and a basement membrane. We will briefly summarize the molecular components forming the tight junction and adherens junction between the vascular endothelial cells and the interaction between endothelial cells and other elements. Figure 1 provides a schematic view of vascular endothelial cells and the major molecules controlling cell shape, cytoskeleton, and mutual cell attachment. VE-cadherin is a homodimeric-binding molecule between endothelial cells that forms a tight border lining. Inside the endothelial cells, VE-cadherin is connected to the alpha/beta-catenin molecule, which, in turn, is linked to the main cytoskeletal element, F-actin. ZO-1 and occludins are the main members of adherens junctions. Brain and retinal vessels have much higher numbers of pericytes than the vessels of other organs [7]. Pericytes maintain the BBB structure, control the contractility of capillaries, and regulate the proliferation, differentiation, and maturation of the component cells—i.e., vascular endothelial cells and oligodendrocytes—through the production of growth factors such as PDGF-BB [3,44,45]. Thus, pericytes play very important roles inside the BBB, orchestrating many cells [46]. To maintain brain function, specific transporters for a diverse range of substances are present on both the luminal and abluminal sides of endothelial cells, pericytes, and astrocytes. Several excellent reviews have been published on this issue [3,47,48,49]. In Figure 1, the factors maintaining the integrity and restriction of BBB permeability are listed, except for MMP-9. HMGB1 is one of the factors inducing an increase in BBB permeability, in association with morphological changes in vascular endothelial cells and pericytes [15,50].

## 2. Stroke and BBB Disruption

Stroke, a condition associated with hypertension, atherosclerosis, and hyperlipidemia, is one of the leading causes of death worldwide. Stroke not only causes mortality in the acute phase but also induces severe neurological complications such as motor paralysis, impairment of higher cognitive functions, and postischemic epilepsy in survivors. However, few effective therapeutics are available for stroke. Stroke actually comprises three kinds of cerebrovascular diseases: brain infarction by thrombosis due to atherosclerosis and atrial fibrillation, hemorrhage by disruption of the vascular wall, and subarachnoid hemorrhage due to a rupture of aneurysm. Thus, the pathophysiological processes that are characteristic of each disease condition are expected to be different, and specific therapeutic strategies must be developed for each type of cerebrovascular disease included under the rubric of stroke.

**Figure 2:**
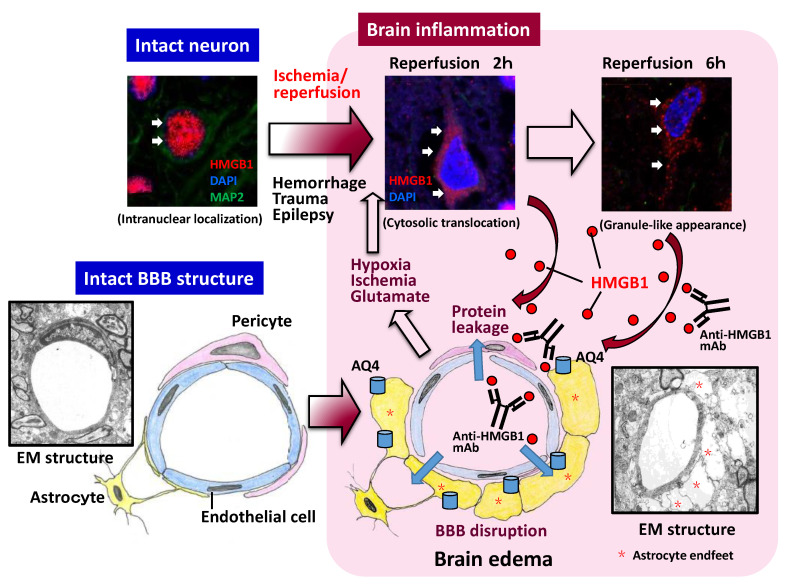
HMGB1 translocation and release after insults and its involvement in BBB disruption. In the upper three squares, typical HMGB1 translocation and release from the nucleus to extracellular space are shown after 2 h of occlusion/reperfusion of the middle cerebral artery. The released HMGB1 then affects the vascular endothelial cells and pericytes, leading to protein leakage and brain edema formation. BBB disruption further promotes HMGB1 translocation. There might be a positive feedback loop between BBB disruption and HMGB1 translocation. The existence of such a loop may be a reason why anti-HMGB1 therapy inhibited BBB disruption and HMGB1 translocation simultaneously. AQ4: aquaporin 4; BBB: blood–brain barrier; EM: electron microscopy; HMGB1: high mobility group box-1; mAb: monoclonal antibody.

Based on their experiments using a middle cerebral artery occlusion/reperfusion (MCAO)-induced injury model in rats, Kim et al. [51] first suggested the relationship between HMGB1 and brain inflammatory injury. They observed that microinjections of short hairpin RNA for HMGB1, which reduced the expression of HMGB1 in the transfected area, inhibited neuronal death after MCAO. They also found that the expression of inflammation-related molecules such as TNF-a, iNOS, IL-1b, and COX-2 was suppressed significantly. Since those factors are thought to originate from the microglia, Kim and colleagues suggested that microglia might be involved in the inflammatory responses mediated by HMGB1 in MCAO rats [51]. Several other groups performed experiments using similar MCAO animal models in rats or mice and found that the neurons in ischemic regions release HMGB1 [14,52,53,54]. HMGB1 translocation in neurons has been shown to occur at an early time point during the ischemic phase [15,52,53,54]. In fact, intranuclear translocation has been observed even 1 h after the start of ischemia [15]. This suggests that HMGB1 translocation is very quick and sensitive to hypoxia and a very early event among the responses after ischemia [15,26] (Figure 2). Such early occurrences of HMGB1 translocation after ischemic insult have also been observed in neonatal rats [55]. In the MCAO model, BBB disruption was evident by electron microscopic observation and by leakage of albumin-binding dye. Using electron microscopy, Zhang et al. [15] showed that the swelling of astrocyte endfeet was remarkable at 3 h after the start of reperfusion around capillary vessels, and the endfeet membrane often detached from the basal membrane of capillary vessels (Figure 2). Dissociation of the tight junction between vascular endothelial cells has also been observed [15]. These are the typical features of BBB disruption under electron microscopy, and all of them were inhibited by the systemic administration of anti-HMGB1 mAb [15]. The morphological changes in BBB structures were consistent with the results of BBB permeability measurement and upregulation of aquaporin-4 on the astrocytic endfeet around capillaries after ischemia [14,15], which were also suppressed by anti-HMGB1 mAb treatment.

Western blotting measurement of HMGB1 content in the core areas of brain ischemia revealed a dramatic decrease in HMGB1 in the core region, while immunohistochemical studies strongly suggested the release of HMGB1 from neurons in severely injured areas [14,15]. It was also reported that an increase in HMGB1 protein was observed in the area of the penumbra after ischemia or hemorrhage [53,56], suggesting that HMGB1 dynamics in such areas differ according to the extent of the neural lesion. Thus, it is likely that the dramatic decrease in HMGB1 was observed in areas with high levels of cell death in the acute phase, whereas the increase in HMGB1 may have been seen in the surviving areas, where the ischemic response included an increase of HMGB1 mRNA.

Zhang et al. [15] detected an increase in HMGB1 levels in both cerebrospinal fluid (CSF) and plasma of brain ischemic animal models. Similar increases in HMGB1 in CSF or plasma have been observed in stroke patients with brain infarction [57], hemorrhage [58], and subarachnoid hemorrhage [59,60]. These findings indicate that some portion of the HMGB1 released from the damaged brain tissue of patients gets into their CSF and plasma, and the degree of increase in HMGB1 probably reflects the severity of their disease states [58,59,60]. In fact, a good correlation between plasma HMGB1 levels and poor outcomes has been reported [61,62]. Therefore, the determination of plasma HMGB1 in patients in the acute phase appears to have prognostic value.

**Figure 3:**
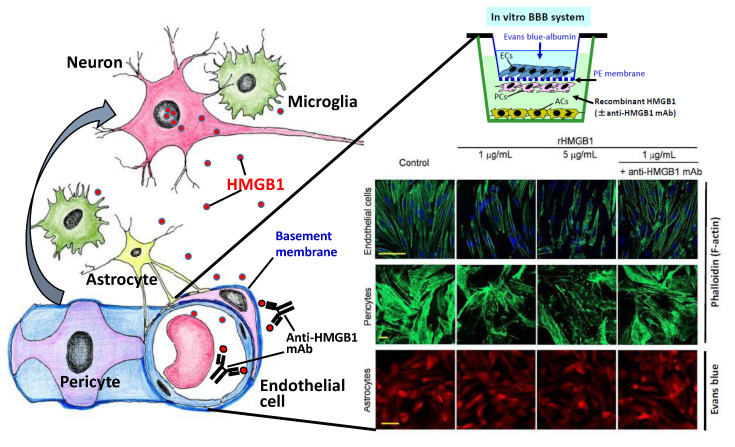
Direct effects of HMGB1 on vascular endothelial cells and pericytes. A rat-reconstituted BBB system composed of vascular endothelial cells, pericytes, and astrocytes (upper right) was used. Recombinant HMGB1 at 1 or 5 mg/mL was added to the brain side (lower chamber), and the incubation was continued for 60 min. After 1 h of stimulation with HMGB1, the cells were fixed with paraformaldehyde. Endothelial cells and pericytes were labeled with Alexa-488-phalloidin, and the astrocytes were labeled with Evans blue. The contractile response was observed in endothelial cells and pericytes, which were inhibited by the presence of specific anti-HMGB1 mAb in the lower chamber (modified from Zhang et al., [15]). BBB: blood–brain barrier; HMGB1: high mobility group box-1; mAb: monoclonal antibody.

**Figure 4:**
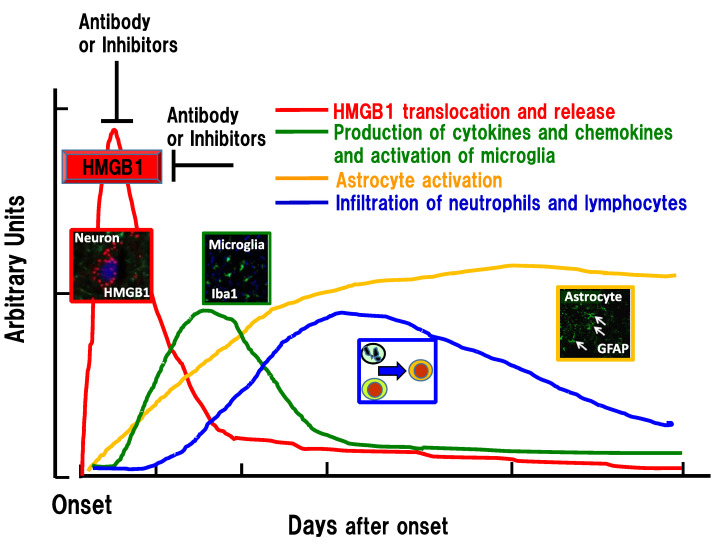
Time-course of inflammation-related events in the penumbra areas after ischemia, hemorrhage, and trauma. HMGB1 translocation and extracellular release form the initial peak to insults, among the known responses, which was followed in succession by microglia activation (cytokine/chemokine production), astrogliosis, and infiltration of immune cells. HMGB1 release may be a very early event that is common to several brain injuries. Inhibition of HMGB1 by the earlier intervention can lead to the diminution of the following inflammatory responses. GFAP: glial fibrillary acidic protein; HMGB1: high mobility group box-1; Iba1: ionized calcium-binding adapter molecule 1.

Intravenous injection of anti-HMGB1 mAb significantly protected against BBB disruption induced by ischemia and hemorrhage in rats [14,15,16] while simultaneously inhibiting the expression of inflammation-related molecules and the activation of microglia. Using a reconstituted BBB in vitro, Zhang et al. [15] demonstrated that the addition of recombinant HMGB1 (rHMGB1) to the lower compartment (the brain parenchymal side) of a Boyden chamber increased the permeability of albumin from the upper compartment (blood side) to the lower (Figure 2). In their experiments, the artificial BBB was composed of endothelial cells, pericytes, and astrocytes. This increase in albumin permeability was associated with the contractile response of vascular endothelial cells and pericytes but not astrocytes [15] (Figure 3). They also revealed that the addition of anti-HMGB1 mAb to the lower compartment, together with rHMGB1, blocked the effects of rHMGB1 on the morphology and function of endothelial cells and pericytes. These results strongly suggest that HMGB1 directly affects the vascular endothelial cells and pericytes in vitro and in vivo. When administered in vivo, anti-HMGB1 mAb may bind to the released HMGB1 and neutralize the action of HMGB1 [14]. This neutralization of HMGB1 probably leads to protection of the BBB, especially when the released HMGB1 exists in close proximity to the vasculatures of the affected brain [25] (Figure 2 and Figure 3). As anti-HMGB1 mAb therapy has been shown to suppress most of the events relevant to brain inflammatory responses after ischemia and hemorrhage—i.e., induction of cytokines, glia activation, and facilitation of immune cell migration [15,16]—it may be possible that the HMGB1 released from neurons and other brain cells functions farther upstream than the other components in the inflammation cascade after insult, leading to a disruption of BBB integrity [26,63] (Figure 4).

Pericyte contraction has been reported to play a very important role in ischemia–reperfusion injury because the delivery of oxygen and nutrients via cerebral microcirculation is fundamental to both metabolic demand and proper cerebral function [64,65]. The demonstration of an HMGB1-induced contractile response in pericytes in vitro [15] strongly supported the notion that HMGB1 released from different cells in the brain directly affects the pericytes and induces the contraction that leads to a reduction of blood flow in microcirculation in vivo [65]. In addition to the contractile response of pericytes to HMGB1, HMGB1 appears to induce an upregulation of the receptors involved in the contraction of smooth muscle cells in the vessels [16]. The HMGB1 released during a stroke may function as one of the factors that provoke the contraction of microvessels [25].

Intriguingly, the systemic injection of anti-HMGB1 mAb has been shown not only to suppress inflammatory responses in the brain and protect BBB integrity but also to inhibit the translocation and release of HMGB1 itself from neurons [14,15,16]. It seems likely that the anti-HMGB1 mAb administered in vivo reaches the capillary vessels first, as described above (Figure 3). Therefore, it is possible that the neutralization of HMGB1 that is close to capillary vessels plays an important role in the initial protection of the BBB, followed by the suppression of the subsequent release of HMGB1 from neurons [25]. In other words, it is suggested that there exists a positive feedback loop between HMGB1 mobilization and brain inflammatory responses, including BBB disruption, in the early phase of the insult [15,16,66]. (Figure 2). In addition to HMGB1 translocation and the release of HMGB1 from neurons, the release of HMGB1 from microglia and astrocytes has been reported to occur in vitro and in vivo, depending on the conditions [16,67,68,69]. Thus, an earlier intervention with anti-HMGB1 mAb to block HMGB1 translocation would be expected to produce a better outcome [15,26]. Concerning the therapeutic time window of anti-HMGB1 mAb, 3–6 h after the start of insult was observed in rat hemorrhage and trauma [16,17]. Therefore, the events triggered by HMGB1 may start very quickly after the start of injury onset because HMGB1 is ubiquitously expressed in almost all cells and appears to be a ready-made mediator (Figure 4). In the future, it will be very important to identify a primary initiator of HMGB1 mobilization and to clarify the molecular mechanism linking tissue injury and HMGB1 release. In macrophages, the balance between acetylation and deacetylation on HMGB1 has been suggested to control the export of HMGB1 from nuclei to the cytosolic compartment [70,71,72]. It should be examined whether the same mechanism could be used to balance the same HMGB1 export in any kind of cells, including neural cells.

Recent work on the translocation of HMGB1 in neurons after ischemic insult indicated that the HMGB1 translocated from nuclei to cytoplasm was colocalized with mitochondria and peroxisomes [73] during the release process. Moreover, HMGB1 was colocalized with Drp-1, a protein involved in the fission of mitochondria, suggesting that HMGB1 also exerts cell-metabolism-modifying effects in these organelles during the translocation process [73].

Matrix metalloproteinases (MMPs) released from pericytes [42,43] have been suggested to be involved in the disruption of the BBB through the digestion of extracellular matrix proteins such as laminin in the basement membrane of capillaries (Figure 1). It is worth mentioning that the mechanism by which HMGB1 upregulates MMP-9 has been investigated in macrophages in peripheral nerve tissue [74].

In addition to neurons and glial cells, vascular endothelial cells may be another origin of extracellular HMGB1 in the brain. Gao et al. [50] reported that a culture of vascular endothelial cells responded to stimuli such as LPS and TNF-a and showed a remarkable translocation of HMGB1 in association with extracellular release without cell death. This release response was almost completely inhibited by the addition of a plasma protein, histidine-rich glycoprotein, at physiological concentration, through the stimulation of C-type lectin receptor-1A on endothelial cells [50]. Based on these findings, it is possible that there might be a receptor-mediated mechanism to control the release of HMGB1 from cells. Further investigations of this issue are warranted.

## 3. Vasospasm after Subarachnoid Hemorrhage 

The treatment for the rupture of intracranial aneurism has been established: clipping of aneurysm or coil embolism. Over the last few decades, however, delayed cerebral ischemia (DCI) due to vasospasm has been one of the major causes of morbidity and mortality in patients with ruptured cerebral aneurysms [46,75,76]. Delayed cerebral vasospasm (DCV) usually develops 7 to 14 days after subarachnoid hemorrhage (SAH). A growing number of reports have suggested possible mechanisms for the vasospasm in experimental animal models [77,78,79]. However, a reliable treatment that controls the vasospasm has not been established [80,81,82]. Numerous clinical trials [83] have evaluated the effects of antagonists for receptors that may be involved in the contractile response of smooth muscle cells in the arterial wall. In addition to these antagonists, calcium channel inhibitors, antiplatelets, statins, and the rho kinase inhibitor fasudil are also candidate drugs for controlling the contractile machinery in smooth muscle cells [78], but there remains insufficient evidence to include any of these agents in the treatment guidelines for vasospasm. Collectively, the above results suggest that a complex mechanism involving inflammatory processes underlies both DCI and vasospasm [80].

In a clinical study, it was shown that HMGB1 levels in the CSF after SAH were correlated with those of IL-6 and TNF-a and that the elevation of HMGB1 in the CSF reflected the level of severity in patients [59]. Thus, considerable evidence suggests the involvement of inflammation in vasospasms after SAH [84,85,86]. Haruma et al. [87] demonstrated that HMGB1 translocation and release, along with marked and persistent vasoconstriction, occurred in smooth muscle cells in the basilar arteries of rats in an autologous blood-injection model of SAH. This vasoconstriction was associated with an increase in expression of several receptor mRNAs in the arterial wall that may be involved in the contractile response of the basilar artery [87]. Post-treatment with anti-HMGB1 mAb efficiently inhibited the vasospasm, in association with a suppression of the mobilization of HMGB1 from smooth muscle cells and upregulation of vasoconstriction-related receptors in the artery [87]. Some inflammatory processes, including the induction of proinflammatory cytokines and the upregulation of RAGE and TLR-4/2, occur in the vascular wall of the basilar artery after SAH, and these were also inhibited by anti-HMGB1 mAb [87] and glycyrrhizin [88], an inhibitor of HMGB1 mobilization. Thus, HMGB1 mobilization, inflammatory responses, and the induction of the arterial contractile state may be interrelated. The strong inhibitory effects of anti-HMGB1 mAb on vasospasms after SAH strongly suggest the involvement of HMGB1 in the induction of the vasocontractile state in the cerebral arteries of the brain after SAH [87]. Since the induction of hyper-responsiveness to thrombin receptor stimulation was reported in a DCI animal model [89,90], the enhancement of responsiveness to thrombin by HMGB1 may be an important factor for the development of vasoconstriction [87,89,90]. Therefore, the mobilization of HMGB1 from vascular smooth muscles may be the event farthest upstream among the cascade of events leading to persistent vasospasm [87,91]. Treatment with anti-HMGB1 mAb may be a preventive therapy for delayed vasospasm in the subacute phase after SAH, blocking the procontractile state of the arterial walls.

## 4. Traumatic Brain Injury and BBB Disruption

Traffic accidents with traumatic brain injury are one of the leading causes of death in younger generations. The main reason for death after traumatic brain injury is brain swelling, in association with brain herniation and hypoperfusion (hypoxia) of brain tissue, leading to the dysregulation of respiratory and cardiovascular centers. Although it has been speculated that the mechanism for brain swelling might be common to different types of brain injuries, it is important to examine in detail whether a release of HMGB1 occurs after traumatic brain injury and, if so, whether the released HMGB1 is involved in the subsequent disruption of the BBB. It was reported that the plasma [92] and CSF levels [93] of HMGB1 increased significantly in patients with brain trauma and that the elevated levels predicted the outcome of patients. Okuma et al. [17] showed that HMGB1 release from neurons occurred within several hours after fluid percussion injury in rats. They mentioned that the immunohistochemical staining pattern of HMGB1 in neurons in traumatic brain injury was not distinguishable from that in the ischemic brain. Anti-HMGB1 mAb therapy maintained the BBB structure under electron microscopy at the affected area and dramatically inhibited brain edema detected by T2-weighted MRI and albumin leakage. Thus, anti-HMGB1 mAb therapy protected BBB disruption morphologically and functionally. As in the case of brain injury induced by ischemia, anti-HMGB1 mAb inhibited the activation of glia cells and the expression of proinflammatory cytokines, as well as inflammation-related factors [14,15]. These effects of anti-HMGB1 mAb on traumatic brain injury were shared by other HMGB1 inhibitors [18,94,95,96], confirming the usefulness of treatments targeting HMGB1 against traumatic brain injury to prevent BBB disruption and brain edema. The analysis of receptors involved in the action of HMGB1 using receptor-knockout mice implied that RAGE and TLR-4 may play roles in the action of HMGB1 [17]. Anti-HMGB1 mAb therapy was applicable to spinal cord injury in a contusion animal model [97,98]. Prior treatment with anti-HMGB1 mAb in mice transplanted with human iPS-derived neuronal stem cells yielded a dramatic improvement in locomotion recovery after spinal cord injury [98]. The combination therapy produced synergistic effects on the contusion-induced spinal cord injury in mice. The beneficial effects of anti-HMGB1 mAb probably come from the inhibition of the initial formation of edema at the injured site and subsequent suppression of glial cell activation [97,98] because there was a therapeutic time window of 6 h for anti-HMGB1 treatment in this animal model [97], which was comparable to that of hemorrhage brain injury in rats [16]. These results strongly suggest that HMGB1 exerts BBB-disrupting effects at the very acute phase of brain injuries [25,26] (Figure 4). A recent study using conditional- and global-knockout mice of HMGB1 implied that HMGB1 may play different roles, intracellularly and extracellularly, in a time-dependent and site-specific manner [99].

## 5. Epilepsy and BBB Disruption

Epilepsy is a neurological disorder with recurrent seizures. Antiepileptic drugs are used to manage most cases of epilepsy. The majority of antiepileptic drugs currently used in clinical settings function by reducing brain excitability or by enhancing inhibition via the manipulation of ion channels. The side effects of antiepileptic drugs are often unavoidable, and about 30% of patients are resistant to current therapies. Therefore, novel cutting edge approaches to epilepsy and epileptogenesis research have been actively sought, with the attendant goal of developing new and more effective drugs [100].

There is increasing evidence suggesting the fundamental roles of inflammatory responses in the initiation of status epilepticus and epileptogenesis [101,102,103,104]. Maroso et al. [105] first demonstrated that kainate injections into the dorsal hippocampus elicited epileptic seizures through the release of HMGB1 from astrocytes, stimulation of TLR-4 in neurons, and inflammatory responses by the activation of microglia [105]. Baram’s group reported that HMGB1 translocation occurred in the amygdala and hippocampus during experimental febrile status epilepticus [106,107]. These findings strongly suggest the involvement of HMGB1 in status epilepticus in experimental animals. Fu et al. [21] observed a marked translocation of HMGB1 in hippocampal and cerebral cortex neurons after systemic injections of pilocarpine and a remarkable inhibition of HMGB1 translocation by systemic injections of anti-HMGB1 mAb, leading to the suppression of status epilepticus. Zhao et al. [108] showed that treatment by systemic injections of anti-HMGB1 mAb produced beneficial effects on four kinds of epileptic animal models, i.e., maximal electroshock-induced, kainate-induced, and picrotoxin-induced convulsions and chronic seizure [108]. It was also demonstrated that superfusion of anti-HMGB1 mAb on the surface of brain slices derived from patients with repeated epilepsy [108] inhibited epileptiform activity in the brain slices. Another group reported that mitochondrial translocation of HMGB1 from nuclei in neurons by epileptic stimuli facilitated neuronal cell death [109,110]. Taking these results together, we speculate that the HMGB1 released from different sources in response to epileptogenic stimuli may mediate the specific processes leading to status epilepticus, with increased neuronal excitability, including glutamate release, upregulation of IL-1b, TNF-a, TLR-4, and disruption of the BBB [21,25,111,112]. Moreover, Zhao et al. [113] demonstrated that neutralization of HMGB1 with mAb inhibited a diazepam-resistant status epilepticus model, and this inhibition was mediated by a TLR-4-dependent pathway. Most of the antiepileptics currently available are activators or inhibitors of ion channels in the plasma membrane. Anti-HMGB1 mAb therapy or HMGB1-release inhibitors may provide an alternative approach to the control of epilepsy and epileptogenesis [114,115]. Nass et al. [116] determined the plasma levels of HMGB1, MMP-9, and ICAM-1 after a single generalized convulsive seizure in epileptic patients. They observed a significant elevation of all these factors and suggested the presence of brain inflammation with BBB disruption even after a single seizure. It is worth mentioning that HMGB1 might be involved in P-glycoprotein expression during status epilepticus, which is related to drug resistance [117,118].

One of the common features of brain injuries induced by ischemia, hemorrhage, trauma, and epilepsy may be BBB dysfunction or disruption. In fact, there is a large body of data from animal model experiments showing an increase in BBB permeability after injury by using different types of tracers of diverse size. Among these tracers, an albumin-binding tracer dye, Evans blue, is commonly used and convenient, and its distribution was demonstrated to merge with the albumin leakage area [17]. The disruption of BBB integrity should involve many processes, including dearrangement of tight junction structures between endothelial cells, lesions of pericytes, digestion of the basement membrane of capillaries, swelling and detachment of astrocyte endfeet from the basement membrane, microglia activation, migration of immune cells from circulation, and release of humoral factors from peripheral tissues. These morphological and functional changes of the BBB should accompany the changes in the gene expression of component cells, cellular signal transduction, and phenotypes of cells. In this respect, little is known about the direct effects of HMGB1 on these component cells in vitro or in vivo. Further studies will be needed to clarify these matters.

## 6. Neurodegenerative Disease and BBB Disruption

Lesions of the cerebral vessels and disruption of the BBB have been suggested to be involved in neurodegenerative diseases, including Alzheimer’s disease, Parkinson’s disease, amyotrophic lateral sclerosis, and multiple sclerosis [3,24,119]. A familial-type mutation of a specific gene may facilitate the aggregation of b-amyloid, a-synuclein, and SOD1. In addition to the impairment of clearance of these aggregates, there might exist inflammation-related responses that induce vascular complications, including BBB disruption [24,119,120].

There is increasing evidence that suggests the involvement of BBB dysfunction or disruption in vascular events, especially in Alzheimer’s disease [120,121,122,123]. The receptor for advanced glycation endproduct (RAGE) was identified as the receptor for AGEs and has been thought to mediate the deleterious effects of AGEs in diabetic patients, with complications of the blood vessels. RAGE functions as a pattern-recognition receptor for a diverse range of factors, and both amyloid b-peptide and HMGB1 are ligands for RAGE. RAGE is expressed on different kinds of cells in the brain, including brain capillaries. An increase in BBB permeability and the leakage of macromolecules from capillaries were observed in the postmortem brains of Alzheimer’s patients [124,125] and an in-vitro BBB model [126,127]. These findings strongly imply that maintenance of the integrity of the BBB is impaired in Alzheimer’s disease. In a mouse model of Alzheimer’s disease, a similar pattern of BBB breakdown was observed before the manifestation of impairment of cognitive functions [128]. Thus, it might be possible that the disruption of the BBB precedes neuronal cell degeneration. In RAGE-knockout mice, the impairment in synaptic functions was reduced compared with that in wild-type mice [129,130], suggesting the involvement of RAGE in the occurrence of symptoms of Alzheimer’s disease. In addition to RAGE, b-amyloid, and LPS, HMGB1 may also play an important role in the activation of RAGE in the brain. It is noteworthy that many reports have suggested the involvement of circulating HMGB1, released from peripheral tissues, in consciousness reduction and impairment in higher cognitive functions. This issue will be described in a later section.

Fujita et al. [131] searched for genes that were upregulated in both the brains of patients with Alzheimer’s disease and a mouse model of Alzheimer’s disease and identified HMGB1 as one such gene. They demonstrated that HMGB1 may play a role in the aggregation of beta-amyloid peptide in Alzheimer’s model mice through the activation of the PKC isoform [131,132]. They also found that chronic treatment with anti-HMGB1 mAb had preventive effects against both beta-amyloid aggregation and cognitive impairment in this animal model. These results imply the involvement of extracellular HMGB1 in the neurogenerative processes in Alzheimer’s disease.

Concerning Parkinson’s disease, systemic administration of anti-HMGB1 mAb inhibited dopaminergic neuronal cell death induced by microinjections of 6-OH dopamine into rat striatum through the suppression of inflammation and the production of reactive oxygen species, in association with protection against BBB disruption [67]. Santoro et al. [133] reported that neutralizing antibodies against HMGB1, as well as glycyrrhizin, inhibited dopamine neuron death induced by MPTP. Taken together, these results suggest that HMGB1 facilitates the cascade of events triggered by dopaminergic neurotoxins. It is worth noting that endogenous intracellular HMGB1 conversely promoted the degradation of a-synuclein by autophagy in the neuroblastoma cell line PC12 [134,135].

There is considerable evidence suggesting the involvement of HMGB1 in the development of multiple sclerosis or neuromyelitis optica [136,137,138,139,140]. However, the specific contribution of HMGB1 to pathogenesis remains to be determined.

Depression, a disorder of mood, is a common disease condition in psychiatric clinics [141]. Although there are several hypotheses concerning the pathophysiology of depression, such as the idea that depression arises from an aminergic neuron disorder, hypercorticosteroidemia due to chronic stress, or insufficiency of BDNF, the mechanistic events in depression remain to be determined. There are increasing reports that suggest that inflammation in the brain is involved in the development of the depressive state [142,143,144,145]. Hisaoka-Nakashima et al. [146] reported that HMGB1-mediated microglial activation induced anxiodepressive-like behaviors in mice with neuropathic pain. This suggests that there is a close relationship between HMGB1-induced brain inflammation and the depressive state. It might be very interesting to pursue this potential association.

## 7. HMGB1 Release from Vascular Endothelial Cells In Vitro

Using a vascular endothelial cell culture, HMGB1 translocation was examined after stimulation with LPS and TNF-a [50]. These stimuli induced the translocation of HMGB1 from nuclei to the extracellular milieu through the cytosolic compartment [50]. The release was dependent on NF-kB activation. It was shown that the recombinant HMGB1 (rHMGB1) induced the upregulation of adhesion molecules ICAM-1 and VCAM-1 on the surface of vascular endothelial cells in the manner of LPS and TNF-a. The upregulation of adhesion molecules was accompanied by the production of the cytokines IL-8, IL-6, and TNF-a by the cells, which was mediated by NF-kB activation. The stimulation by rHMGB1 was also associated with the upregulation of HMGB1 receptors, namely, toll-like receptor-2/4 and RAGE. Therefore, there might be a self-activation mechanism in endothelial cells that is mediated by HMGB1. It has been speculated that disorders of vascular endothelial cells play a role in severe systemic inflammation, especially under septic conditions. Accordingly, HMGB1 release from vascular endothelial cells may have multiple effects that contribute to the activation of the endothelial cells themselves. It should be pointed out that a plasma protein, histidine-rich glycoprotein [147], antagonized the effects of HMGB1 on vascular endothelial cells [50]. This relationship suggests the fine regulation of the interface between plasma and vascular endothelial cells [50]. Together, these results suggest that HMGB1 induced multiple effects that directly stimulated vascular endothelial cells in the same manner as LPS and TNF-a.

## 8. The Relationship between Peripheral Inflammation and BBB

As mentioned in the section on stroke, the plasma or CSF levels of HMGB1 increase significantly depending on the severity of brain injury. This increase may promote lung inflammation because it is well known that a portion of patients with stroke develop pneumonia concomitantly [148,149,150]. On the other hand, it may be that the inverse sequence occurs, with peripheral inflammation influencing the CNS [151,152]. A rat model of postischemic infection treated with LPS exhibited an exacerbation of infarct formation [153], indicating amplification of brain inflammation through the systemic load of endotoxins. In humans, it was demonstrated that peripheral surgery induced BBB disruption [154,155]. Influenza pneumonia can induce BBB breakdown by the load of endotoxins, which may be mediated by HMGB1 [156]. Thus, there are many reports indicating a relationship between peripheral inflammation and BBB disruption [157]. Among cases of peripheral inflammation, the most dramatic may be those with septic encephalopathy, which is characterized by an evident increase in the capillary permeability of brain vessels and brain swelling, leading to a reduction in consciousness [38].

In COVID-19, delirium and encephalopathy have been reported to be observed in more than 80% of patients [158]. Another group determined the plasma levels of S100 A8/9 and HMGB1 in the circulation and found a correlation between the elevation of S100A8/9 and HMGB1 and the clinical severity of patients [159]. Thus, it is likely that the elevation of these DAMPs, reflecting peripheral inflammation, may, in part, contribute to the occurrence of CNS symptoms through the effects on the BBB and blood cells [160].

HMGB1 binds to lipopolysaccharides, and the HMGB1–LPS complex appears to be transported into lysosomes in hepatocytes, leading to the activation of caspase-11 and IL-1 secretions [39]. It is noteworthy that HMGB1 plays proinflammatory roles through plural pathways.

## 9. Therapeutic Methods Targeting HMGB1

There are several classes of drugs that have therapeutic potential for inhibiting the effects of HMGB1 in the brain. Table 1 summarizes the different approaches and their mechanisms: box-A proteins, antagonists for HMGB1 receptors; neutralizing mAbs, binding aptamers or peptides; siRNA for HMGB1; inhibitors of HMGB1 secretion. Concerning the inhibitors of HMGB1 secretion, many kinds of herb-derived substances have been reported, including glycyrrhizin. This strongly suggests that the anti-inflammatory effects of some of the prescriptions in traditional medicine may be, in part, ascribable to the presence of HMGB1 inhibitors in the complex compositions.

## 10. Conclusions

**Figure 5:**
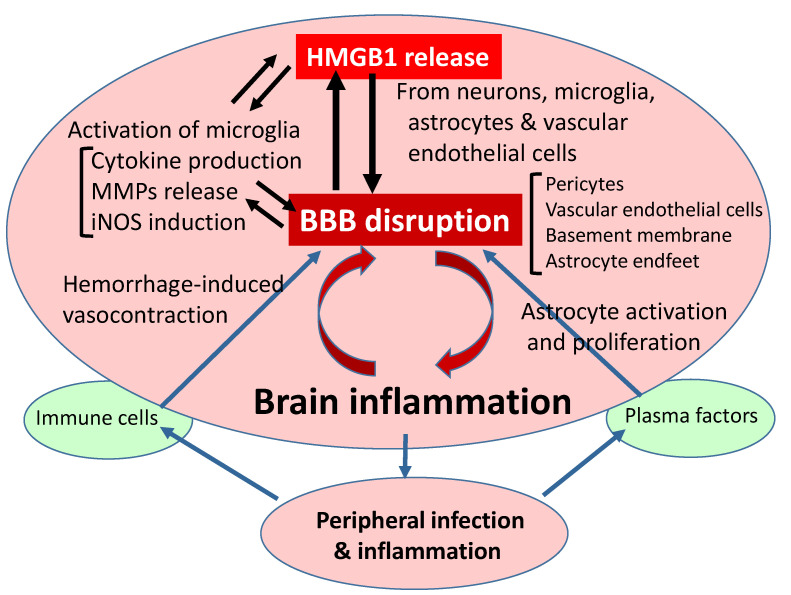
Flow diagram of relationship between HMGB1 release and BBB disruption. As described in the text, HMGB1 release is common to different kinds of injuries induced by ischemia/reperfusion, hemorrhage, trauma, and epilepsy, leading to BBB disruption through direct and indirect pathways. The peripheral inflammation caused by infection may exacerbate BBB disruption. Conversely, brain injuries may induce a vulnerability to infection in patients. BBB: blood–brain barrier; HMGB1: high mobility group box-1; iNOS: inducible nitric oxide synthase; MMPs: matrix metallo proteinases.

Inflammatory responses after cerebral ischemia, hemorrhage, trauma, or epilepsy are closely associated with BBB dysfunction and disruption. During the inflammatory processes, HMGB1 may be released extracellularly from neurons, glia, or endothelial cells, depending on the situation (Figure 5). Anti-HMGB1 mAb therapy and HMGB1 inhibitors were demonstrated to be beneficial within a specific therapeutic time window for different kinds of inflammatory diseases in animal models, especially those containing BBB disruption. In other words, the extracellular release of HMGB1 may play very important roles in triggering the initial inflammatory responses through the stimulation of multiple receptors, leading to BBB disruption. Therefore, therapies with anti-HMGB1 mAb and inhibitors of HMGB1 release may provide novel approaches to the treatment of brain inflammatory diseases associated with BBB disruption. A clear understanding of the mutual relationship between peripheral and CNS inflammation and the roles of HMGB1 in mediating these processes must await further studies.

## Figures and Tables

**Table 1:** Effects of different HMGB1 inhibitors on brain inflammation and BBB disruption.

Thrapeutics	Models	Animals, Route	Reference
Box-A protein	Meningitis	Mice, i.p.	Hohne et al. [161]
	TBI	Mice, i.v.	Yang et al. [162]
Anti-HMGB1 mAb	MCAO/R	Rats, i.v.	Liu et al. [14]
	TBI	Rats, i.v.	Okuma et al. [17]
	Hemorrhage	Rats, i.v.	Wang et al. [16]
	TSCI	Mice, i.p.	Uezono et al. [98]
	SAH	Rats, i.v.	Haruma et al. [87]
	Epilepsy	Mice, i.v.	Fu et al. [21]
siRNA	MCAO/R	Rats, i.c.i	Kim et al. [51]
	MCAO/R	Rats, i.n.	Kim et al. [163]
Binding peptide	MCAO/R	Rats, i.n.	Kim et al. [164]
Ethyl pyruvate	MCAO/R	Rats, i.p.	Yu et al. [165]
	SC ischemia	Rabbits, i.v.	Wang et al. [166]
	TBI	Rats, i.p.	Su et al. [95]
	Hemorrhage	Rats, i.p.	Lei et al. [56]
Release inhibitors			
Glyccyrrhizin	MCAO/R	Rats, i.v.	Kim et al. [167]
	I/R	Mice, i.v.	Ni et al. [168]
	TBI	Rats, Mice, i.v.	Okuma et al. [18]
	TSCI	Rats, i.v.	Gong et al. [169]
	Hemorrhage	Rats, i.p.	Ohnishi et al. [170]
	SAH	Rats, i.p	Li et al. [88]
Tanshinone II	pMCAO	Rats, i.p.	Wang et al. [171]
Berberine	MCAO/R	Mice, i.g.	Zhu et al. [172]
Ginsenosides	MCAO/R	Rats, i.g.	Xie et al. [173]
Ursolic acid	MCAO/R	Rats, i.g.	Wang et al. [174]
Others			
Omega-3 PUFA	TBI	Rats, i.p.	Chen et al. [175]
Soluble thrombomodulin	MCAO/R	Mice, i.v.	Nakamura et al. [176]

MCAO/R: middle cerebral artery occlusion and reperfusion; pMCAO: permanent middle cerebral artery occlusion; TBI: traumatic brain injury; TSCI: traumatic spinal cord injury; SAH: subarachnoid hemorrhage; SC ischemia: spinal cord ischemia; i.p.: intraperitoneal; i.v.: intravenous; i.n.: intranasal; i.g.: intragastric; PUFA: polyunsaturated fatty acid.

## Abbreviations

BBB: blood–brain barrier; CNS: central nervous system; CBF: cerebral blood flow; CSF: cerebrospinal fluid; DAMP: damage-associated molecular pattern; DCI; delayed cerebral ischemia; DCV: delayed cerebral vasospasm; HMGB1: high mobility group box-1; mAb: monoclonal antibody; MCAO: middle cerebral artery occlusion; NVU: neurovascular unit; RAGE: receptor for advanced glycation endproduct; rHMGB1: recombinant HMGB1; SAH: subarachnoid hemorrhage.
